# Kavalactone Kawain Impedes Urothelial Tumorigenesis in UPII-Mutant Ha-Ras Mice via Inhibition of mTOR Signaling and Alteration of Cancer Metabolism

**DOI:** 10.3390/molecules28041666

**Published:** 2023-02-09

**Authors:** Zhongbo Liu, Liankun Song, Jun Xie, Xue-Ru Wu, Greg E. Gin, Beverly Wang, Edward Uchio, Xiaolin Zi

**Affiliations:** 1 Department of Urology, University of California Irvine, Orange, CA 92868, USA; 2 Veterans Affairs Long Beach Healthcare System, Long Beach, CA 90822, USA; 3 Department of Urology, NYU School of Medicine, New York, NY 10016, USA; 4 Veterans Affairs New York Harbor Healthcare System, New York, NY 10010, USA; 5 Department of Pathology and Laboratory Medicine, University of California, Irvine, Orange, CA 92868, USA; 6 Chao Family Comprehensive Cancer Center, University of California Irvine, Orange, CA 92868, USA

**Keywords:** Kawain, mTOR, chemoprevention, bladder cancer

## Abstract

UPII-mutant Ha-ras transgenic mice develop urothelial hyperplasia and low-grade papillary carcinoma, which mimics human non-muscle invasive bladder cancer (NMIBC). We investigated the effects and mechanisms of kawain, a main kavalactone in the kava plant, on oncogenic Ha-ras-driven urothelial carcinoma in these mice. The mice were fed at six weeks of age with vehicle control or kawain (6 g/kg) formulated food for approximately five months. Seventy-eight percent of the mice or more fed with kawain food survived more than six months of age, whereas only 32% control food-fed male mice survived, (*p* = 0.0082). The mean wet bladder weights (a surrogate for tumor burden) of UPII-mutant Ha-ras transgenic mice with kawain diet was decreased by approximately 56% compared to those fed with the control diet (*p* = 0.035). The kawain diet also significantly reduced the occurrence of hydronephrosis and hematuria in UPII-mutant Ha-ras transgenic mice. Histological examination and immunohistochemistry analysis revealed that vehicle control-treated mice displayed more urothelial carcinoma and Ki67-positive cells in the bladder compared to kawain treated mice. Global metabolic profiling of bladder tumor samples from mice fed with kawain food showed significantly more enrichment of serotonin and less abundance of xylulose, prostaglandin A2, D2 and E2 compared to those from control diet-fed mice, suggesting decreased shunting of glucose to the pentose phosphate pathway (PPP) and reduced inflammation. In addition, kawain selectively inhibited the growth of human bladder cancer cell lines with a significant suppression of 4E-BP1 expression and rpS6 phosphorylation. These observations indicate a potential impact of kawain consumption on bladder cancer prevention by rewiring the metabolic programs of the tumor cells.

## 1. Introduction

Human urinary bladder cancer is the fourth in men and the twelfth in women among the most prevalent cancers in the United States (US) [1]. Up to 80% of bladder cancers are early stage, non-muscle invasive bladder cancer (NMIBC). These patients often experience high recurrence rates (75–85%) after their initial diagnoses, which require long-term follow-up with repeated invasive diagnostic and treatment procedures. Because of the lifetime needs for surveillance and treatment of recurrent tumors, and the combined high cost of complications with treatments, human urinary bladder cancer is a major public health liability in the US [2]. Approximately $3.7 billion (2001 US dollars) is paid out in the US each year on bladder cancer treatment [3]. Therefore, prevention through lowering bladder cancer occurrence and recurrence is potentially an effective approach for mitigating the burden of bladder cancer management.

Over-activation of Ha-ras via point mutation, overexpression, or FGF receptor 3 activation has been described in 70–90% of low-grade, papillary, and NMIBC [4,5]. Therefore, the Ha-ras pathway represents a major target for the prevention and treatment of this type of bladder cancer. While male heterozygous UPII-mutant Ha-ras transgenic mice develop only simple urothelial hyperplasia over a long period of time, the homozygous mice develop marked urothelial hyperplasia at one month, nodular hyperplasia between two and three months, multifocal and papillary bladder tumors by four months, and die by five and six months due to urinary obstruction and nephropathy [6]. Tumors in homozygous mice also were frequently observed in the renal pelvis and ureters [6]. The male homozygous UPII-mutant Ha-ras transgenic model allows us to study the effect of an agent on the survival (one of the most important endpoints for cancer prevention and treatment) of bladder tumor-bearing mice in a feasible way. Molecularly, the UPII-mutant Ha-ras transgenic model mirrors high mTOR activity, which presents in approximately 70% of human urinary bladder cancer [6,7,8,9]. The mTOR pathway regulates aerobic glycolysis, and then rewires the metabolic programs of the tumor cells to promote cell proliferation [10]. Therefore, the male homozygous UPII-mutant Ha-ras transgenic model is currently the most suitable model for examining the preventive and treatment effects of novel agents on bladder cancer metabolism and recurrence progression [7,8].

Kava (*Piper methysticum Forst*) is an ancient and social drink, and has been connected to remarkably low incidences of smoking-related cancers, including lung and bladder cancers, in several small islands of the South Pacific region where there are many smokers and kava drinkers among the populations [11,12]. In addition, potent anti-carcinogenic and anti-tumor activities of kava root extracts have recently been shown in some studies [12,13,14,15]. To facilitate the further mechanistic and toxicological evaluation of a chemopreventive candidate, we have chosen to focus on studying pure kava components for cancer prevention [16,17,18,19]. Kawain is a major kavalactone from kava root extracts [20]. Here, we have presented data for the first time to show that dietary kawain exhibits a strong anti-tumor effect in the UPII-mutant Ha-ras model and increases the survival of non-muscle invasive urothelial tumor-bearing mice, as well as delays the progression from hyperplasia to urothelial papillary carcinoma and reduces urothelial cell carcinoma (UCC) induced hydronephrosis and hematuria. Dietary kawain also significantly reduces in vivo bladder cancer cell proliferation and stimulates apoptosis, which is coupled with reduced mTOR signaling and altered cancer metabolisms.

## 2. Results

### 2.1. Kawain-Formulated Diet Reduces Tumor Burden and Increases the Survival of UPII-Mutant Ha-Ras Transgenic Mice

**Figure 1:**
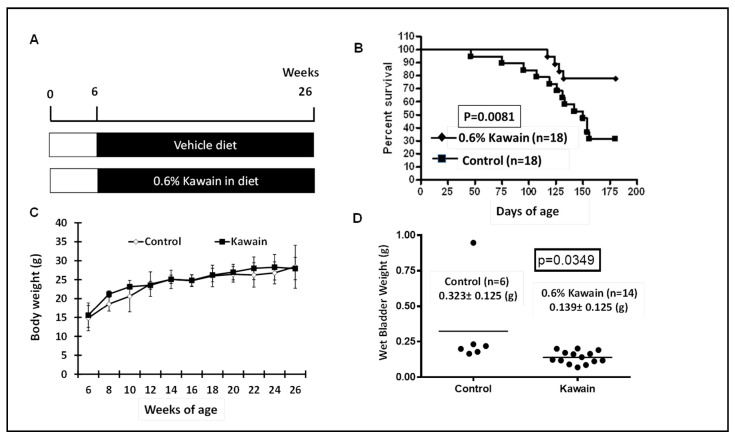
Dietary feeding of kawain reduces tumor burden and increases the survival of UPII-mutant Ha-ras mice. (**A**) An experimental protocol for evaluating chemopreventive efficacies of dietary kawain in the UPII-mutant Ha-ras model. (**B**) The survival curves of male mice fed with indicated diet beginning at 6 weeks of age until 6 months of age or up to their death. (**C**,**D**) The mean body weight documented every week and bladder weights at the end of the experiment of male UPII mutant Ha-ras mice fed with indicated diet as shown in the figure.

Genotyped mice with bladder specific expression of mutant Ha-ras at the age of six weeks were randomly assigned with control diet or with 0.6% kawain in diet. The mice were continuously fed with these experimental diets for 20 weeks or until their death due to development of UCC (Figure 1A). Food consumption, body weight and health conditions of the mice were also monitored weekly. Figure 1B shows that 4 out of 18 (78%) mice in the kawain diet group survived more than six months compared to 12 out of 18 (33%) mice in the control diet group, and that the mice in the kawain diet group lived much longer (*p* = 0.0081). Body weights are similar between the two groups (Figure 1C). The mean of wet bladder weights in the kawain diet group were also reduced by 56% compared to that of mice fed with control diet (Figure 1D, *p* = 0.0349). 

### 2.2. Kawain Diet Inhibits the Pathological Progression of Hyperplasia to Papillary Carcinoma

**Figure 2:**
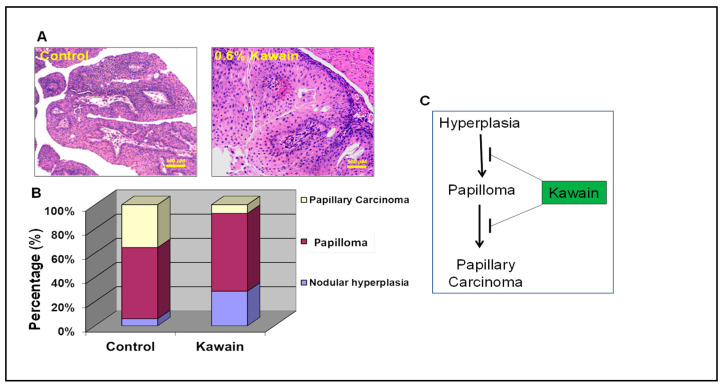
**Kawain diet inhibits UCC evolution from hyperplasia to papillary carcinoma.** (**A**) Microscopic examination of bladders of male UPII mutant Ha-ras mice fed with indicated experiment diet for five months. Magnification: 100×. (**B**) Percentages of papillary hyperplasia, nodular hyperplasia, papilloma, and papillary carcinoma of male UPII mutant Ha-ras mice fed with indicated diet for five months as shown in the figure. (**C**) Schematic presentation of the effect of kawain diet on the pathological progression of urothelial cell carcinoma.

H&E staining and histological analysis revealed that bladder and urothelial tissues from mice fed with 0.6% kawain diet exhibited more nodular hyperplasia and less papillary carcinoma in comparison with those mice fed with the control diet (Figure 2A,B). These results suggested that the kawain diet inhibits the pathological progression of hyperplasia to papillary carcinoma in UPII-mutant Ha-ras transgenic mice (Figure 2C).

### 2.3. Kawain Diet Reduces the Incidence of Hydronephrosis and Hematouria

**Figure 3:**
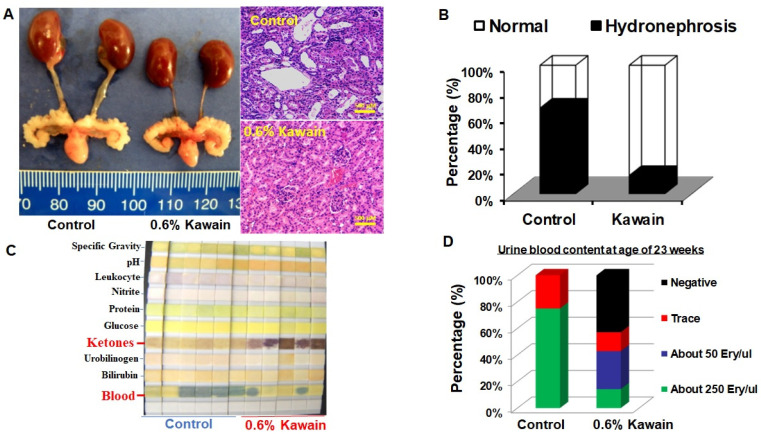
**Kawain diet reduces the occurrences of hydronephrosis and hematuria.** (**A**) macrographic and micrographic examination of enlarged kidneys and ureters. (**B**) Percentages of mice with hydronephrosis were shown in mice fed with the indicated diet. (**C**) Photographs of dipsticks after dipping with urine from male UPII-mutant Ha-ras mice fed with indicated diet at the age of 23 weeks. (**D**) Percentages of mice with hematuria shown in control and kawain diet fed mice.

Urothelial papilloma and papillary carcinoma are also detected in the renal pelvis or the ureter in the UPII mutant Ha-ras model, which often results in hydronephrosis and irreversible damage of kidney function [6,7,8]. This model also images human upper tract UCC. Four out of six (66.7%) examined mice in the control diet group exhibited obvious hydronephrosis, whereas only 2 out of 14 (14.3%) examined mice in the kawain diet group were observed with hydronephrosis (Figure 3A,B). This result indicates that kawain diet decreased the incidence of hydronephrosis by 52.4%. 

In addition, we collected urine from control- and kawain-diet-fed mice at age of 23 weeks and performed urine analysis using urinalysis test strips from Roche Diagnostics, as reported in our previous publications [7,8]. Seventy-five percent of control-diet-fed mice were detected with more than 250 erythrocytes/microliter in urine, whereas only 14.3% (1/7) of kawain-diet-fed male mice were found with more than 250 erythrocytes/microliter at the age of 23 weeks (Figure 3C,D). The majority of kawain-diet-fed mice were detected with ketone body in their urine, but this was absent in urine from control-diet-fed mice, suggesting that the kawain diet may have effects on fat catabolism in the UPII mutant Ha-ras model [21].

### 2.4. Kawain Diet Decreases Cell Proliferation and Increases Apoptosis In Vivo

**Figure 4:**
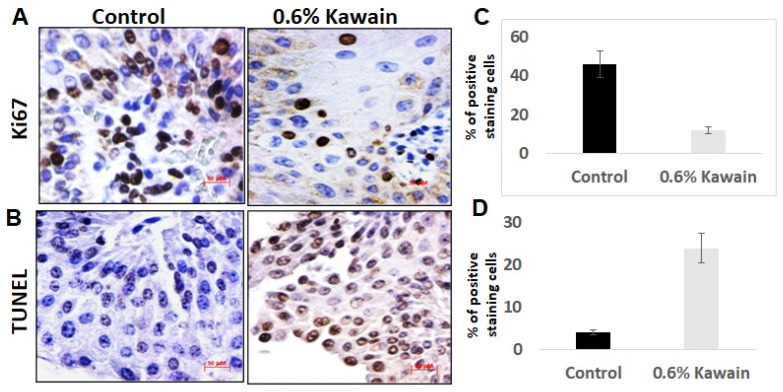
**Kawain diet decreases cell proliferation and promotes apoptosis in tumor tissues.** (**A**,**B**) Brown-colored positive cells for Ki-67 (proliferative marker) and TUNEL (apoptotic marker) were shown at ×200 magnification from representative IHC-stained bladder tumor sections randomly selected from six mice fed with indicated diet. (**C**,**D**) Quantitative results of positive-staining cells form 12 fields in each section from each group. Mean ± SD in histography was shown and Student’s *t*-test used. All *p*-values are <0.01.

Approximately 46% ± 3.3% Ki-67-positive staining cells were detected by IHC analysis in bladder tumor tissues from the mice fed with control diet compared to 12.0 ± 2.6% Ki-67-positive staining cells from mice fed with kawain diet (Figure 4A,B, *p* < 0.01). In contrast, more TUNEL-positive cells were present in tumor tissues from mice administrated with kawain diet compared to those with control diet (Figure 4C,D; kawain vs. control groups: 24.2 ± 4.7% and 4 ± 0.8%, *p* < 0.01). These results supported that the antitumor effects of kawain diet were through both inhibition of proliferation and induction of apoptosis. 

### 2.5. Kawain Selectively Inhibits the Growth of Bladder Cancer Cell Lines and TSC1 Positive Cells, and Kawain Diet Attenuates the mTOR Signaling In Vivo

**Figure 5:**
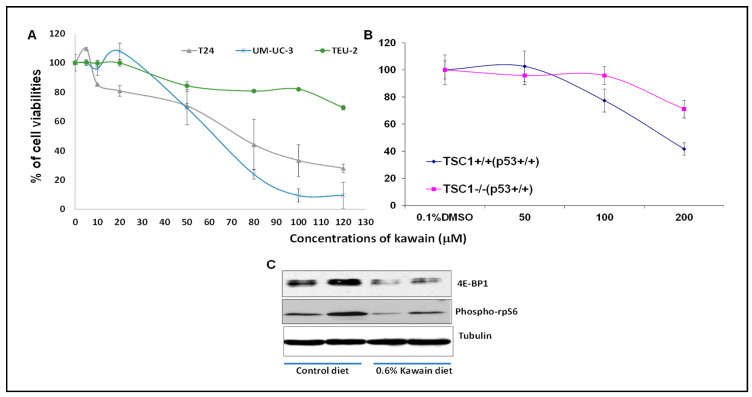
**The growth inhibitory efficacies of kawain are related to its inhibition of the mTOR signaling.** (**A**) Growth inhibitory efficacies of kawain on the growth of T24, UMUC3 and Teu-2 cells after treatments with 0.1% DMSO or indicated concentrations of kawain for 72 h. (**B**) TSC1 knockout MEFs are more resistant to kawain’s cytotoxic effects compared to TSC1 wild-type ones. (**C**) The protein expression levels of the mTOR pathway effectors: 4E-BP1 and phosph-rpS6, in bladder tumor tissues from mice fed with indicated diet were analyzed by Western blotting.

Figure 5A shows that kawain reduced cell viabilities of bladder cancer cell lines: T24 and UMUC-3 in a dose-dependent manner, whereas non-malignant bladder urothelial TEU-2 cells were resistant to the cytotoxicity of kawain. TSC1 deletion primary mouse embryo fibroblasts (TSC1-/- MEFs) were also resistant to growth inhibitory effects of kawain compared to their wild-type MEFs, suggesting that the expression of TSC1 in MEFs may be partially required for the growth inhibitory efficacy of kawain. Further Western blotting analysis of bladder tumor tissues revealed that dietary kawain resulted in a reduced protein expression of 4E-BP1 and phosphorylation of rpS6. Multiple 4E-BP1 protein bands were detected and the slower migrated band of 4E-BP1 in the blot represent its phosphorylation forms as suggested in published studies [7], suggesting that kawain diet inhibited both 4E-BP1 phosphorylation and the expression of total protein. This result indicates that kawain diet inhibits the mTOR signaling in vivo. 

### 2.6. Kawain Diet Changes the Metabolism of UPII-Ha-Ras Bladder Tumors

**Figure 6:**
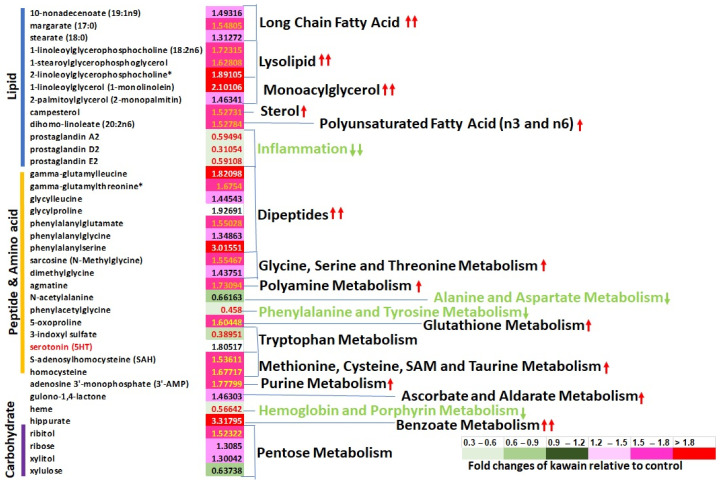
**Kawain diet induces metabolic changes in bladder tumor tissues of the UPII Ha-ras mice.** Differential levels of metabolites of lipid, peptide and amino acid, and carbohydrates of bladder tumor tissues from seven pairs of control- vs. kawain-treated mice were detected by global biochemical profiling using liquid chromatography-tandem mass spectroscopy (LC-MS). The numbers of biochemicals that accomplished statistical significance (*p* ≤ 0.05) were shown and presented as a fold change of kawain diet relative to control diet.

Next, we performed metabolomic profiling of tumor tissues for a total of 342 metabolites. Fifty (36 up and 14 down) of them were founded to be statistically significantly altered in tumor tissues from kawain-treated mice in comparison with control-diet-fed mice, with statistical correction for the multiple comparisons and an estimate of the false discovery rate (q-value). Lysolipids and several monoacylglycerols and long-chain fatty acids were more abundant in tumor tissues from the kawain-diet-fed mice compared to the control, suggesting increased lipase activity (Figure 6). Likewise, many dipeptides were more abundant with kawain treatment, suggesting increased protein catabolism, but urea was less abundant, suggesting incomplete oxidation of amino acids released from protein catabolism (Figure 6). Serotonin, a neurotransmitter generated from tryptophan, homocysteine, and S-adenosylhomocysteine (SAH), a substrate for methylation reactions, were also enriched with kawain treatment, although other tryptophan metabolites, such as 3-indoxyl sulfate, were less abundant with kawain treatment. Kawain-treated samples had lower levels of prostaglandins A2, D2 and E2 when compared with control samples (Figure 6). Nucleotide monophosphates (3′AMP) was elevated with kawain treatment relative to control samples. With kawain treatment, the pentose phosphate pathway (PPP) side product xylulose were less abundant (Figure 6), suggesting decreased shunting of glucose to the PPP.

## 3. Materials and Methods

### 3.1. Chemopreventive Efficay of of Dietary Kawain on Mutant Ha-Ras-Driven Urothelial Carcinogenesis

Cohorts of UPII-mutant Ha-ras mice were generated as described previously [6,7,8] and were provided (a) control diet (AIN93M diet) and (b) 0.6% kawain [0.6% kawain (*w*/*w*) in AIN-93M diet] (Dyets, Inc., Bethlehem, PA, USA) [8]. Each group contained 18 mice. These treatments started at four weeks of age and allow a one-week equilibration period after weaning. Comparable initial body weight was achieved in each group through a randomization process. Groups of survived mice were terminated at six months of age by CO_2_ asphyxiation. We have recorded: (1) body weight and food consumption weekly, (2) time to death or sacrifice, (3) organ weight (heart, lung, liver prostate thymus, kidney, etc.) at the end of the experiment, (4) tumor burden (bladder and ureteral weight), (5) pathological changes: simple hyperplasia, nodular hyperplasia, and low-grade papillary carcinoma, and (6) obstructive uropathy [6,7,8]. The chemopreventive efficacies were comprehensively analyzed by comparing bladder and ureteral weight, survival time, pathological grade, incidence of hydronephrosis, and time to hematuria to the control groups. The approved protocol by UCI was followed (protocol #:2004-2540) for animal care and all experimental procedures. 

### 3.2. Histology and Immunohistochemistry (IHC) Analyses

Paraffin-embedded blocks were cut in 5 μm thick slides for standard hematoxylin and eosin (H&E) staining. Histological examination was performed as described in detail in our previous publications [6,7,8].

In addition, proliferation marker Ki67 and apoptosis were evaluated on bladder tumors using anti-Ki-67 antibody (Abcam, 1:800, Waltham, MA, USA) and the DeadEnd Colorimetric TUNEL assay kit (Promega, Madison, WI, USA), respectively, with appropriate positive and negative controls as described previously [7,8]. Percentages of positive staining cells were double-blindly obtained by counting the total number of cells from 12 randomly selected, ×200 magnified fields.

### 3.3. In Vitro Cell Proliferation Assay 

T24 and UMUC3 cell lines from ATCC were cultured in McCoy 5A growth medium and Eagle’s Minimum Essential Medium (EMEM), respectively, with 10% fetal bovine serum (FBS) added. TEU-2 cells are immortalized, non-malignant bladder epithelial cells [18]. Known species of mycoplasma contamination were tested in these cell lines using a kit from Lonza Inc (Houston, TX, USA). Cells were plated at a density of 2 × 10^4^ in 24-well plates and 24 h later, treated with a variety of kawain concentrations or 0.1% dimethyl sulfoxide (DMSO) for 72 h. Cell proliferation was evaluated with 3-4,5-Dimethylthiazol-2-yl (MTT) assay as described in our previous publications [16,22]. Percentage ratios of vehicle-treated controls and the kawain dose-response curves were calculated. 

### 3.4. Western Blotting Analysis 

The treated frozen tumor tissues were homogenized and extracted in Radio-Immunoprecipitation Assay (RIPA) buffer contained a protease inhibitor cocktail. Western blotting analysis was performed to examine the effects of kawain treatment on expression levels of 4E-BP1 and phosphor-rpS6 [18]. β-tubulin was used as a loading control. 

### 3.5. Metabolomic Profiling

Freshly frozen bladder tumor samples from both control and 0.6% kawain diet fed mice as described above were processed in the automated MicroLab STAR^®^ system by Metabolon, Inc. (Durham, NC, USA). For quality control in the extraction process, a recovery standard was added before the first step purposes. A total of five fractions were analyzed by ultra-high-performance liquid chromatography mass spectrometry (UPLC-MS/MS) with positive ion mode electrospray ionization, UPLC-MS/MS with negative ion mode electrospray ionization, UPLC-MS/MS polar platform (negative ionization), and gas chromatography (GC)-MS, respectively, with a backup. The QC and curation processes were carried out for data normalization and correcting variation from instrument interday tuning differences. System artifacts, misassignments, and background noise were also removed. Peaks were quantified using area-under-the-curve. The normalized metabolite abundance was log-transformed if required.

### 3.6. Statistical Evaluation

Data were presented as means ± SD. We performed a Student *t*-test to detect the significance of observed differences between vehicle control and kawain diet groups. Survival curves were plotted and analyzed by the Kaplan–Meier test. *p* ≤ 0.05 was needed as significant. GraphPad Prism 8 Software (Irvine, CA, USA) and Excel were used for statistical analyses.

## 4. Discussion

Kava has been a social drink among the South Pacific Island residents for thousands of years due to its soothing properties [23]. Kavalactones are the key bioactive elements in kava root extracts that are responsible for their stress reducing and anti-anxiety effects, and kawain is one of the main kavalactones [20]. Recent studies by us and other investigators have shown that kava root extracts have chemopreventive potential against tumorigenesis of many cancers, including prostate, lung and colon cancers [8,11,12,13,14,15,16,17,18,19,20,22]. However, there are very few studies on the anti-tumor effects of kawain as a pure component, and no animal experimental studies of the chemopreventive properties of kawain toward human urinary bladder cancer have been reported. Therefore, we are the first to demonstrate that a kawain-supplemented diet significantly reduces the tumor burden, delays the pathological progression of hyperplasia to papillary carcinoma, increases the survival of mice bearing mutant Ha-ras-driven urothelial tumors. Our results support that kawain is a potential candidate for further testing its chemopreventive potential in human urinary bladder cancer. 

We also demonstrate that a kawain diet alters the tumor metabolisms through increasing fat and protein catabolism and decreasing inflammatory metabolites, as well as lessening shunting of glucose to the PPP. Kawain selectively inhibits the growth of bladder cancer cell lines over non-malignant urothelial cells and downregulates mTOR signaling. Tumor growth and progression depends on the reprogramming of metabolisms in both tumor and tumor microenvironment [24,25]. Recently, Dr. Xue-ru Wu and his associates have demonstrated that increased glycolysis by enolase-1 or pyruvate kinase 2 (PKM2) can promote tumorigenesis in mutant Ha-ras-driven urothelial carcinoma [26,27]. In this study, we observe that the PPP side product xylulose was reduced in the bladder tumors from kawain-diet fed mice, which suggests decreased shunting of glucose to the PPP. Whether the expression or function of enolase-1 or PKM2 contribute to the anti-tumor efficacy of dietary kawain remains unclear. However, these results indicate that further understanding the cooperation between oncogene function and metabolic adoption or addiction in mutant Ha-ras-driven urothelial carcinoma would bring new opportunities for bladder cancer prevention and combination therapies.

We also observed that the kawain diet resulted in a significant increase in serotonin and a decrease in 3-indoxyl sulfate in bladder tumors. Both are metabolites of tryptophan and associated with relieving anxiety [28]. The degradation of serotonin is mainly catalyzed by the mitochondrial enzyme monoamine oxidase A [29]. This result aligns well with mood-bettering effects of kava root extracts, and our recent report that kawain is a cell active and weak inhibitor of monoamine oxidase A [14]. Evidence continues to accumulate that chronic psychological stress affects cancer growth, metastasis and cellular aging, and is thought to be a risk factor at least for specific types of cancers [30,31,32]. Therefore, further understanding the role of kawain on the stress interaction with cancer may provide novel supplementary strategies for cancer management.

Lysolipids, generated by phospholipase activity toward membrane phospholipids, were elevated with kawain treatments, suggesting increased lipase activity [33]. Also consistent with increased lipase activity, several monoacylglycerols and long-chain fatty acids were more abundant with kawain treatment [34]. Lysolipids may be generated for membrane remodeling as a part of autophagy [35]. Our recent publication has shown that yangonin, a kavalactone with a similar chemical structure to kawain, induces autophagy via inhibition of the mTOR pathway in bladder cancer cells [36] The phosphoinositide 3 kinase (PI3K)/protein kinase B (AKT)/mTOR pathway is the most frequently activated pathway in human urinary cancer. Up to 70% of urothelial tumors were found with hyper-activation of the PI3K/AKT/mTOR pathway [9]. mTOR signaling regulates cell growth and metabolism, including lipid, peptide/amino acid, and carbohydrate metabolism. On the contrary, metabolites, such as amino acids, activate mTOR signaling. Therefore, further studies are warranted to investigate the complex relationship among kavalactones, mTOR signaling, cell metabolism and autophagy.

Tang et al. [37] reported a strong anti-inflammatory effect of kawain against collagen antibody-induced arthritis in mice. Kawain treatment of RAW264.7 monocyte/macrophage-like cells also resulted in a reduction of lipopolysaccharide-induced tumor necrosis factor alpha, interleukin 27, and monokine induced by gamma interferon secretion via inhibition of MyD88, Akt and lipopolysaccharide-induced tumor necrosis factor-alpha factor [37]. Inflammation stimulates production of prostaglandins [38]. These signaling molecules are derived from PLA2 activity against membrane lipids that release unsaturated C20 fatty acids, which are further metabolized by COX-2 to produce prostaglandins [38]. In this study, prostaglandins A2, D2 and E2 were all significantly lower in tumor tissues from mice fed with the kawain diet. A molecular docking and QSAR classification-based virtual screening have identified kawain as a potential COX-2 inhibitor from a Natural Product Library [39]. These studies together have provided solid evidence for the anti-inflammation effect of kawain. Therefore, further studies will be performed to confirm the inhibitory effect of kawain on COX2 activity.

In conclusion, our results indicate that kawain could be further developed as potential chemopreventive agent for reducing the frequency of NMIBC recurrence and preventing its progression. For a future study, global metabolic profiles of plasma could be used as a readout of the cumulative effects of kawain treatments in other organs. Additionally, as a main site of coordination of metabolic signals and xenobiotic metabolism, liver profiling could provide new insight into the mechanisms of action of kawain treatments.

## Data Availability

Not applicable.
